# IgLON5 Regulates the Adhesion and Differentiation of Myoblasts

**DOI:** 10.3390/cells10020417

**Published:** 2021-02-17

**Authors:** Jeong Ho Lim, Mirza Masroor Ali Beg, Khurshid Ahmad, Sibhghatulla Shaikh, Syed Sayeed Ahmad, Hee Jin Chun, Dukhwan Choi, Woo-Jong Lee, Jun-O Jin, Jihoe Kim, Arif Tasleem Jan, Eun Ju Lee, Inho Choi

**Affiliations:** 1 Department of Medical Biotechnology, Yeungnam University, Gyeongsan 38541, Korea; lim2249@naver.com (J.H.L.); mirzamasroor1986@gmail.com (M.M.A.B.); sibhghat.88@gmail.com (S.S.); sayeedahmad4@gmail.com (S.S.A.); po98053@gmail.com (H.J.C.); apdltkd@naver.com (D.C.); jinjo@yu.ac.kr (J.-O.J.); kimjihoe@ynu.ac.kr (J.K.); 2 Research Institute of Cell Culture, Yeungnam University, Gyeongsan 38541, Korea; ahmadkhursheed2008@gmail.com; 3 Biomedical Manufacturing Technology Center, Korea Institute of Industrial Technology, Yeongcheon 38822, Korea; wjlee@kitech.re.kr; 4 School of Biosciences and Biotechnology, Baba Ghulam Shah Badshah University, Rajouri 185234, India; atasleem@gmail.com

**Keywords:** extracellular matrix, IgLON5, muscle stem (satellite) cell, myoblast, myogenesis, skeletal muscle

## Abstract

IgLON5 is a cell adhesion protein belonging to the immunoglobulin superfamily and has important cellular functions. The objective of this study was to determine the role played by IgLON5 during myogenesis. We found IgLON5 expression progressively increased in C2C12 myoblasts during transition from the adhesion to differentiation stage. IgLON5 knockdown (IgLON5_kd_) cells exhibited reduced cell adhesion, myotube formation, and maturation and reduced expressions of different types of genes, including those coding for extracellular matrix (ECM) components (COL1a1, FMOD, DPT, THBS1), cell membrane proteins (ITM2a, CDH15), and cytoskeletal protein (WASP). Furthermore, decreased IgLON5 expression in FMOD_kd_, DPT_kd_, COL1a1_kd_, and ITM2a_kd_ cells suggested that IgLON5 and these genes mutually control gene expression during myogenesis. IgLON5 immunoneutralization resulted in significant reduction in the protein level of myogenic markers (MYOD, MYOG, MYL2). IgLON5 expression was higher in the CTX-treated gastrocnemius mice muscles (day 7), which confirmed increase expression of IgLON5 during muscle. Collectively, these results suggest IgLON5 plays an important role in myogenesis, muscle regeneration, and that proteins in ECM and myoblast membranes form an interactive network that establishes an essential microenvironment that ensures muscle stem cell survival.

## 1. Introduction

Skeletal muscles are contractile tissues constitute ~40% of total body weight [1,2] and consist of cylindrical multinucleated myofibers and resident stem cells, i.e., muscle stem cells (MSCs; satellite cells) [3,4]. These cells have a regeneration ability that gradually declines with age [5,6]. Myogenesis involves cell cycle arrest, myogenic activation, cell alignment, multiple cell fusion, an increase in nuclear size, and the peripheral localization of nuclei [7].

MSCs are located between basal lamina and sarcolemma [5,6], which provides anatomical and functional stability to skeletal muscle [8]. MSCs actively control myofiber growth, and progression, which are regulated by myogenic regulatory factors (myoblast determination protein; MYOD, myogenin; MYOG, and muscle regulatory factor 4; MRF4) [9].

Collagens, laminins, and fibronectin (FN) are glycoproteins and major components of extracellular matrix (ECM) and adhere to cellular proteoglycans (PGs). Glycoproteins also support skeletal muscle integrity, facilitate architectural support, and participate in myogenesis regulatory signaling [10]. ECM participates in various cell-signaling processes and serves as a modulator of growth factors during cell growth [10,11]. Furthermore, PGs like fibromodulin (FMOD) participate in ECM assembly [3] and are profoundly associated with myogenesis [3,8].

FMOD was found to be elevated during bovine MSCs differentiation as determined by a comparative DNA microarray analysis [4], and a microarray analysis of FMOD knockdown C2C12 myoblast cells showed down regulation of integral membrane protein 2A (Itm2A), type I collagen, dermatopontin (DPT) [12], Itm2A, and DPT participate in muscle differentiation and myotube formation [13,14]. Type I collagen and non-collagenous matrix protein DPT [14,15] provide tensile and mechanical strengths to tissues [14]. Furthermore, it was reported thrombospondin-1 (THBS1) expression was suppressed in DPT knockdown C2C12 cells at the mRNA and protein levels [14]. THBS1, a multidomain calcium-binding glycoprotein [16], is highly expressed during muscle development following injury [17].

In addition, to explore the role of FMOD in myoblast differentiation, FMOD knockdown resulted in the downregulation of IgLON5 (immunoglobulin-like cell adhesion molecule 5) [8]. The IgLON family (a component of the immunoglobulin superfamily (IgSF) [18]) is composed of a large group of cell-surface proteins, such as OPCML (opioid-binding cell adhesion molecule, IgLON1), NTM (neurotrimin, IgLON2), LSAMP (limbic system-associated membrane protein, IgLON3), NEGR1 (neuronal growth regulator 1, IgLON4), and IgLON5 (immunoglobulin-like cell adhesion molecule 5) are widely expressed in the central nervous system [19]. IgLON protein family members possess three Ig domains and a glycosylphosphatidylinositol (GPI) anchor [20,21], which facilitate them to attach to lipid rafts of cytoplasmic membranes [22], and communicate as homo- and heterodimers [23]. IgLON5 has a 336 amino acid chain and shows the closest similarity with OPCML (50%), NTM (48–49%), and LSAMP (46–47%), and the least similarity with NEGR1 (41%). IgLON protein family members participate in neuronal pathfinding, neurite growth, and synapse formation during brain growth [24] and are importantly associated with encephalopathies, sleep dysfunction, chronic neurodegenerative diseases, and cell-surface related autoimmune disorders [25,26], and have also been involved in the pathogeneses of autism spectrum diseases and solid tumors [27].

However, as mentioned above, IgLON5 has only been studied in the nervous system and its activities at a molecular level have not been elucidated. Therefore, as an extension of our previous studies, we aimed to determine whether IgLON5 is responsible for myogenesis and if so, to investigate the mechanism involved. Our results demonstrate that interactions between IgLON5 and various genes are involved in the initial adhesion and differentiation of C2C12 cells and suggest IgLON5 is involved in muscle regeneration in mice.

## 2. Materials and Methods

### 2.1. Cell Culture

Murine C2C12 myoblast cells (Korean Cell Line Bank, Seoul, Korea) were cultured in growth media [DMEM (Dulbecco’s modified Eagle’s medium) supplemented with 10% FBS (fetal bovine serum) and 1% P/S (penicillin/streptomycin; all from HyClone Laboratories, South Logan, UT, USA)] in a humidified 5% CO_2_ incubator at 37 °C. After cells had reached 90% confluency, growth media was switched to differentiation media (DMEM + 2% FBS +1% P/S), and cells were cultured for 0, 2, 4, or 6 days, during which media were changed daily.

### 2.2. Neutralization of IgLON5

After reaching 90% cell confluency, growth media was switched to differentiation media supplemented with 5 μg/mL IgLON5 antibody (Bioss, Woburn, MA, USA) and cells were incubated for a further 2 or 4 days.

### 2.3. Gene Knockdown

Cells were grown until 30% confluent in growth media and transfected with 1 ng of IgLON5, FMOD, DPT, COL1a1, or ITM2a shRNA or a scrambled vector containing the GFP gene using transfection reagents and media (Santa Cruz Biotechnology, CA, USA). Transfected cells were selected with 2 μg/mL puromycin (Santa Cruz Biotechnology, CA, USA) for 3 days and knockdown efficiencies were confirmed by comparing IgLON5, FMOD, DPT, COL1a1, and ITM2a mRNA levels in knockdown cells with those in scramble vector-transfected cells. shRNA sequence information is provided in Table S1.

### 2.4. Cell Adhesion and Proliferation Assays

The equal numbers of IgLON5_wt_ (IgLON5 wild type) and IgLON5_kd_ (IgLON5 knockdown) cells were cultured in growth media for 3 h (for the adhesion study) or 3 days (for the proliferation study). For MTT assays, cells were then incubated in CellTiter 96^®^ AQueous One Solution Reagent (Promega, Madison, WI, USA) for 1 h at 37 °C. Absorbance was measured at 490 nm using a microplate reader (Biotek Synergy H1, Winooski, VT, USA).

### 2.5. Co-Culture of IgLON5_wt_ and IgLON5_kd_ Cells

IgLON5_wt_ and IgLON5_kd_ cells were trypsinized, counted, and equal numbers of IgLON5_wt_ and IgLON5_kd_ cells were co-cultured in growth medium until 90% confluent and then in differentiation medium for 4 days.

### 2.6. Cell Adhesion Analysis Using Centrifugation

IgLON5_wt_ or IgLON5_kd_ cells were cultured in growth medium for 2 or 4 days (until 30% or 100% confluent) or in differentiation medium for 4 days. Then, plates were filled with growth or differentiation media and sealed with microplate sealing tape. Plates were placed inverted, and centrifuged at different ‘g’ values in the centrifuge with a swinging bucket rotor for 5 min. After removing media, MTT assays were performed as above to measure the adhesion of cells.

### 2.7. Fusion and Maturation Indices

Fusion indices were determined as previously described [14]. In brief, cells were fixed with methanol and stained with 0.04% Giemsa G250 (Sigma Aldrich, St. Louis, MO, USA) and then washed with PBS. Images were taken randomly at three different places in dishes (300×). Total numbers of nuclei in cells and numbers of nuclei in myotubes were counted, and fusion indices were calculated by expressing numbers of nuclei in myotubes as radio of total numbers of nuclei. Myotube indices were calculated as the ratio of five or more nuclei in one myotube among the total nuclei.

### 2.8. Cell Cycle Analysis

IgLON5_wt_ and IgLON5_kd_ cells were cultured in growth medium for 3 days, trypsinized, washed twice with ice-cold PBS, and left overnight in 70% ethanol at −20 °C. The ethanol was then removed by centrifugation, and cells were washed twice with ice-cold PBS. Cells were then treated with cell cycle reagent (Merck Millipore, Darmstadt, Germany) for 30 min in the dark at room temperature and performed with flow cytometry (EasyCyte5HT; Merck Millipore, MA, USA). The measurement data were analyzed by Guava^®^ InCyte software (Merck Millipore, MA, USA).

### 2.9. Metabolite Analysis

IgLON5_wt_ and IgLON5_kd_ cells were cultured in growth medium for 1 or 3 days, respectively. Cultured media were collected and an enzyme colorimetric method was performed to estimate glucose, lactate, and NH_3_ concentrations using a bioanalyzer (Cedex; Roche Diagnostics, Indianapolis, IN, USA).

### 2.10. Animal Experiment

C57BL/6 male mice (Daehan Biolink, Dae-Jeon, South Korea) were maintained in a temperature-controlled room under a 12 h light/12 h dark cycle with free access to water and food. A normal diet containing 4.0% (*w*/*w*) total fat (RodentNIH-31 Open Formula Auto; Zeigler Bros., Inc., Gardners, PA, USA) was supplied for 16 or 26 weeks when gastrocnemius (gas) muscle tissues were collected, fixed, and stored at −80 °C. As previously described [14], mice were anesthetized with avertin, and then 100 μL of 10 μM cardiotoxin (CTX) or PBS (control) was injected into the left or right gas muscles, respectively. Muscles were collected 7 and 14 days after injection for further analysis. All experiments were carried out in accord with the guidelines issued by the Yeungnam University Institutional Animal Care and Use Committee (AEC2015-006).

### 2.11. Immunocytochemistry

Immunocytochemistry was performed using antibodies against MYOD, MYOG, MYL2 (Myosin light chain 2), FMOD, DPT, COL1a1, ITM2a, WASP (Wiskott–Aldrich syndrome protein), CDH15 (Cadherin 15), and IgLON5. C2C12 cells were grown on glass-bottomed dishes, washed twice with PBS, and fixed with 4% formaldehyde for 10 min. After permeabilization with 0.2% Triton X-100 (Sigma Aldrich, St. Louis, MO, USA), cells were incubated with primary antibodies [MYOD, MYOG, FMOD, DPT, ITM2a, WASP, CDH15 (1:50, Santa Cruz Biotechnology), MYL2, COL1a1 (1:50, Abcam, Cambridge, MA, USA)], or IgLON5 (1:50, Bioss, Bioss Antibodies Inc. Massachusetts, USA) overnight at 4 °C in a humid environment. Secondary antibodies (1:100, Alexa Fluor 488 or 594 goat anti-rabbit or mouse antibody SFX kit; Molecular Probes, Invitrogen, Carlsbad, CA, USA) were applied for 1 h at room temperature in the dark. After rinsing cells twice with PBS, nuclei were counterstained with DAPI (4′, 6-diamidino-2-phenylindole; 1:1000, Sigma Aldrich). Fluorescence imaging was performed with Nikon Eclipse TE2000-U inverted fluorescence phase contrast microscope, using objective Nikon Plan Fluor ELWD 20X 0.45 (Nikon, Melville, NY, USA) and a ProgRes C3 CCD Digital Microscope Camera (Jenoptik, Jena, Germany) controlled by i-solution software (IMT, Dae-Jeon, South Korea).

### 2.12. Total RNA Extraction, cDNA Synthesis, and Real-Time RT-PCR

Total RNA was extracted from cells using Trizol^®^ reagent (Invitrogen, CA, USA), according to the manufacturer’s instructions, and a high capacity cDNA reverse transcription kit (Applied Biosystems, Foster City, CA, USA) was used to synthesize 1st strain cDNA. Briefly, 2 μg of total RNA in a 20 μL reaction mixture containing random hexamers and reverse transcriptase was used to synthesize 1st strand cDNA using the following schedule; 25 °C for 10 min, 37 °C for 120 min, and 85 °C for 5 min. Gene expressions were analyzed using cDNA (2 μL) and gene-specific primers (10 pmole, 2 μL) and a 7500 real-time PCR system using Power SYBR Green PCR Master Mix (Thermo Fisher Scientific, MA, USA). All reactions were performed in triplicate, and relative amounts of gene expressions normalized vs. controls were calculated using 2^−∆Ct^, where ∆C_t_= C_t_ gene−C_t_ control. Glyceraldehyde 3-phosphate dehydrogenase was used as an internal control. The primers used are detailed in Table S2.

### 2.13. Western Blot Analysis

C2C12 cells and muscle tissues were lysed using RIPA buffer containing 1% protease inhibitor cocktail (Thermo Fisher Scientific, Waltham, MA, USA), and total protein concentrations were quantified by Bradford assay. Proteins (50 μg) were electrophoresed in 4–12% gradient SDS-PAGE (Invitrogen, CA, USA) and transferred to PVDF membranes (Millipore, Billerica, MA, USA), which were then blocked with 3% skim milk or BSA (bovine serum albumin) in TBS (Tris-buffered saline) containing 0.1% Tween 20 for 1 hr and incubated with protein-specific primary antibodies [MYOD (1:400), MYOG (1:400), MYL2 (1:400), IgLON5 (1:1000), FMOD (1:400), DPT (1:400), COL1a1 (1:400), IMT2a (1:400), THBS1 (1:400), WASP (1:400), CDH15 (1:400), and β-actin (1:2000)] in TBS containing 1% skim milk or BSA, overnight at 4 °C. Blots were subsequently washed and incubated with horseradish peroxidase-conjugated secondary antibodies (goat anti-mouse or anti-rabbit; Santa Cruz Biotechnology) at room temperature for 2 h and then reacted with Super Signal West Pico Chemiluminescent Substrate (Thermo Fisher Scientific, MA, USA).

### 2.14. Immunohistochemistry and Immunofluorescence

Immunohistochemistry was performed as previously described [28]. Briefly, paraffin-embedded gas muscle tissues (non-injected, CTX injected) were deparaffinized and hydrated using a xylene (Junsei, Tokyo, Japan) and ethanol (Merck Millipore, Massachusetts, United States) series, and treated with 0.3% H_2_O_2_/methanol to block endogenous peroxidase activity. Sections were then either stained with H&E (hematoxylin and eosin) or blocked with 1% normal goat serum (KPL, Gaithersburg, MD, USA), incubated with IgLON5 antibodies (1:50) at 4 °C overnight, and then incubated with secondary antibody horseradish peroxidase-conjugated (1:100). Signals were visualized by adding diaminobenzidine and hydrogen peroxide. Negative controls were worked up by omitting the primary antibody. Stained sections were observed under a light microscope (Leica, Wetzlar, Germany).

Immunofluorescence was deparaffinized and hydrated in the same process as immunohistochemistry, and blocking was performed with 1% goat serum. Incubated with IgLON5 and PAX7 (Santa Cruz Biotechnology, CA, USA) antibody (1:50) at 4 °C overnight, and then incubated with secondary antibody (1:100, Alexa Fluor 488 or 594 goat anti-rabbit or mouse antibody SFX kit). Fluorescence images were taken in the same way as previously mentioned immunocytochemistry.

### 2.15. Statistical Analysis

Mean normalized expression was compared using Tukey’s studentized range (honestly significant difference) to identify significant gene expressional differences. The *p*-values of ≤0.05 were considered statistically significant. Real-time RT-PCR data were analyzed by one-way ANOVA using PROC GLM in SAS ver. 9.0 (SAS Institute, Cary, NC, USA).

## 3. Results

### 3.1. IgLON5 Regulated Myoblast Differentiation

**Figure 1:**
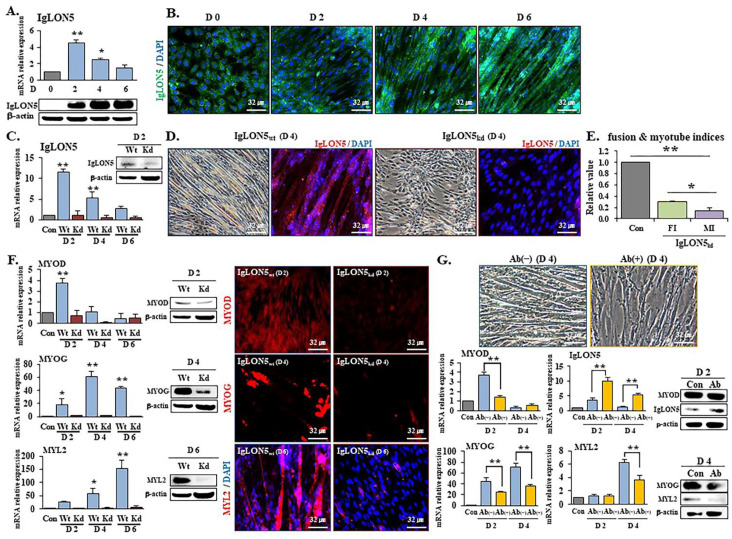
IgLON5 expression, knockdown, and immunoneutralization in C2C12 cells. (**A**) IgLON5 mRNA and protein expressions on differentiation days 0, 2, 4, and 6 were determined by real-time RT-PCR and Western blot, respectively. (**B**) IgLON5 protein localization as determined by immunocytochemistry on differentiation days 0, 2, 4, and 6. (**C**) IgLON5 mRNA expression as determined by real-time RT-PCR and IgLON5 protein expression by Western blot in IgLON5_wt_ and IgLON5_kd_ cells on differentiation days 2, 4, and 6 versus IgLON5_wt_ cells on differentiation day 0. (**D**) IgLON5 protein localization in IgLON5_wt_ and IgLON5_kd_ cells determined by immunocytochemistry on differentiation day 4. (**E**) Fusion and myotube indices (FI, MI) determined by Giemsa staining of IgLON5_kd_ cells on differentiation day 4 as compared with IgLON5_wt_ cells. (**F**) MYOD, MYOG, and MYL2 mRNA and protein expressions by real-time RT-PCR and Western blot, and protein localizations by immunocytochemistry in IgLON5_wt_ and IgLON5_kd_ cells on differentiation days 2, 4, and 6 versus IgLON5_wt_ cells on differentiation day 0. (**G**) Morphologies of cells treated without Ab(−) or with Ab(+) IgLON5 antibody on differentiation day 4. MYOD, MYOG, MYL2, and IgLON5 mRNA and protein expressions in cells treated without Ab(−) or with Ab(+) IgLON5 antibody on differentiation days 2 and 4 versus differentiation day 0. Wt indicates transfected with scrambled vector. Means ± SD (n ≥ 3). * *p* < 0.05, ** *p* < 0.01. (Scale bar, 32 µm).

IgLON5 mRNA expression was elevated during the early cell differentiation stage (day 2) and then gradually decreased (days 4 and 6). However, IgLON5 protein expression progressively increased during differentiation (Figure 1A,B). mRNA and protein expressions of IgLON5 and myogenic marker genes (MYOD, MYOG, and MYL2) (Figure 1C,F and Figure S1A,B), and fusion indices (FIs) and myotube indices [29] (Figure 1D,E) were lower for IgLON5_kd_ cells than IgLON5_wt_ cells. The effect of IgLON5 knockdown was checked using an IgLON5 specific antibody at different time points during differentiation. The results obtained, including myotube formation findings, were similar to those obtained for IgLON5_kd_ cells. Myogenic marker genes were downregulated in antibody-treated cells, though MYOD protein levels were not significantly different on day 2 and IgLON5 protein levels were increased (Figure 1G). These results suggest IgLON5 controlled C2C12 differentiation and myotube formation, that myotube maturation was accomplished by regulating myogenic genes, and that IgLON5 mRNA and protein expressions were increased to compensate for the blocking of IgLON5 protein in cell membranes.

### 3.2. IgLON5 Expression and Myotube Formation

**Figure 2:**
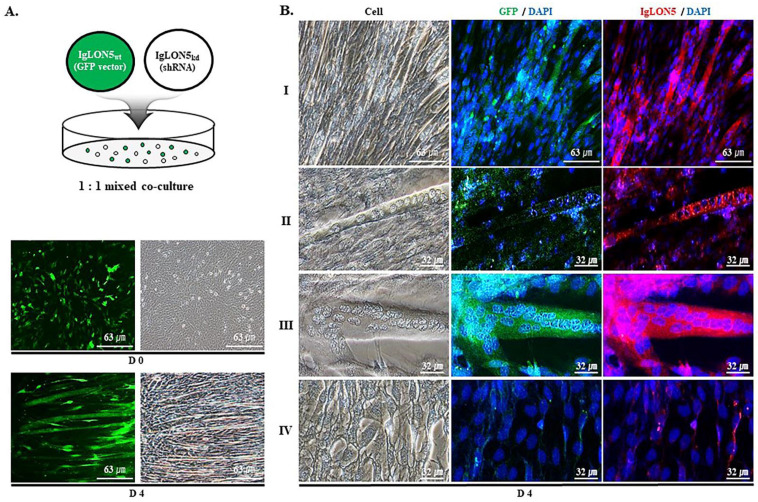
IgLON5 expression during myotube formation by C2C12 cells. (**A**) An equal number of IgLON5_wt_ and IgLON5_kd_ cells were co-incubated in differentiation media for 0 days (D 0) and 4 days (D 4), respectively. Only IgLON5_wt_ cells containing GFP vector emitted green fluorescence on differentiation days 0 and 4 (Scale bar, 63 µm). (**B**) Observations of green and red fluorescence from GFP (IgLON5_wt_ cells) and IgLON5 cells on differentiation day 4. Pictures were taken in the typical area (**I**) or in areas where narrower myotubes were formed [30], wider myotubes were formed (**III**), or where no myotubes formed (**IV**), respectively (Scale bar, 63 or 32 µm). IgLON5_wt_ indicates cells transfected with a scrambled vector.

Equal numbers of IgLON5_wt_ cells transfected with the GFP gene via the transfection vector and IgLON5_kd_ (lacking the GFP gene) cells carrying three different shRNA constructs targeting IgLON5 mRNA were mixed and co-cultured. IgLON5_wt_ cells were evenly distributed as cells reached ~90% confluence (day 0) and a strong GFP signal was observed where myotubes formed (day 4), which indicated that most myotubes were derived from IgLON5_wt_ cells (Figure 2A). Only GFP expressing cells (IgLON5_wt_) formed myotubes and expressed IgLON5 (Figure 2B,I). Furthermore, GFP and IgLON5 were less expressed in narrow myotubes, but strong and distinct expression was observed in thick myotubes (Figure 2B, II and III). GFP and IgLON5 were rarely observed where myotubes were not formed (Figure 2B, IV). These results imply that myotubes were formed by cells normally expressing IgLON5, which suggests IgLON5 promoted myotube maturation by thickening myotubes.

### 3.3. The roles of IgLON5 during Adhesion and Proliferation

**Figure 3:**
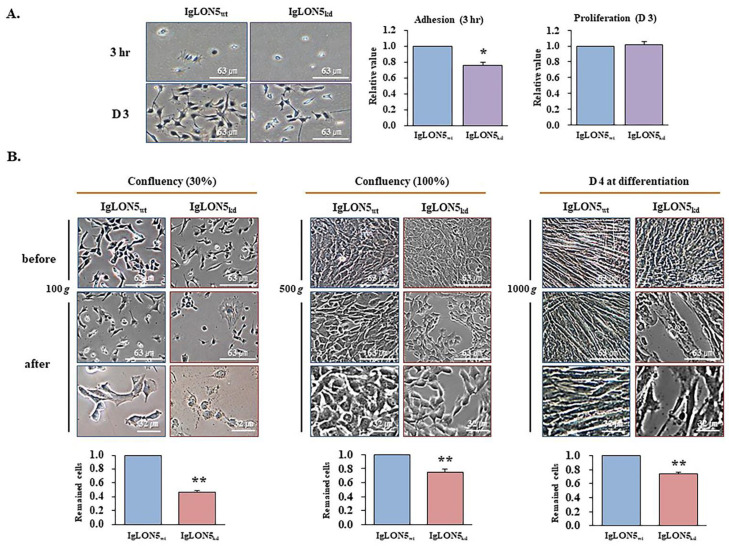
Adhesion, proliferation, and differentiation of IgLON5_wt_ and IgLON5_kd_ C2C12 cells. (**A**) IgLON5_wt_ and IgLON5_kd_ cells were allowed to attach to culture dishes for 3 h or 3 days (Scale bar, 63 µm). Cell adhesion was expressed as the relative values of adhered cells, determined by MTT assay in IgLON5_wt_ and IgLON5_kd_ cells at 3 h. Cell proliferation at day 3 was expressed as the relative values of the cells as determined by MTT assay in IgLON5_wt_ and IgLON5_kd_ cells at 3 days after normalization by the relative values of adhered cells at 3 hr. (**B**) IgLON5_wt_ and IgLON5_kd_ cells were cultured until 30% or 100% confluent, or for 4 days from the differentiation commencement (before centrifugation), and then sealed cell culture dishes were placed inverted and centrifuged using a swinging bucket rotor at 100, 500, or 1000× *g* (after centrifugation, scale bar, 63 or 32 µm). Relative values of the remained cell numbers in IgLON5_wt_ and IgLON5_kd_ cells after centrifugation, measured by MTT assay and normalized with the each initial cell numbers before centrifugation, respectively (graphs at the bottom). IgLON5_wt_ indicates cell transfected with scrambled vector. Means ± SD (n ≥ 3). * *p* < 0.05, ** *p* < 0.01.

IgLON5_wt_ and IgLON5_kd_ cells were cultured in growth media for 3 h to investigate adhesion or for 3 days to investigate proliferation. After 3 hrs of incubation, adhesion by IgLON5_kd_ cells was significantly less than for IgLON5_wt_ cells. However, 3 days incubation, no significant difference was observed between cell proliferation (Figure 3A). When IgLON5_wt_ and IgLON5_kd_ cell cycles and metabolites in culture media were analyzed on day 3, no significant difference was observed between the two cells types in terms of the percentages of cells in the G_0_/G_1_, S, G_2_/M phases (Figure S2A) and metabolite (glucose, lactate, NH_3_) concentrations in cell culture media (Figure S2B). These findings revealed IgLON5 plays an important role in cell adhesion during myoblast attachment, and did not affect the cell cycle and cellular metabolism.

The role played by IgLON5 in C2C12 cell adhesion during proliferation and differentiation was investigated by exposing adherent cells to different ‘g’ forces during centrifugation. IgLON5_wt_ and IgLON5_kd_ cells reached 30%, 100% confluency and on differentiation day 4, and then cell culture dishes were inverted and centrifuged at 100× *g* (30% confluency), 500× *g* (100% confluency), or 1000× *g* (differentiation day 4), respectively. We found IgLON5_kd_ cells adhered less than IgLON5_wt_ cells in all three cell states. Furthermore, 30% confluent IgLON5_wt_ cells appeared normal after centrifugation whereas IgLON5_kd_ cells were abnormally shaped. Major changes were observed on differentiation day 4, as IgLON5_wt_ cells maintained the myotube shape whereas IgLON5_kd_ cells did not. These observations suggest that IgLON5 helps maintain C2C12 cell integrity by increasing cell-to-cell and cell-to-surface adhesion during proliferation and differentiation (Figure 3B).

### 3.4. Gene Expression Profiles in IgLON5_wt_ and IgLON5_kd_ Cells

**Figure 4:**
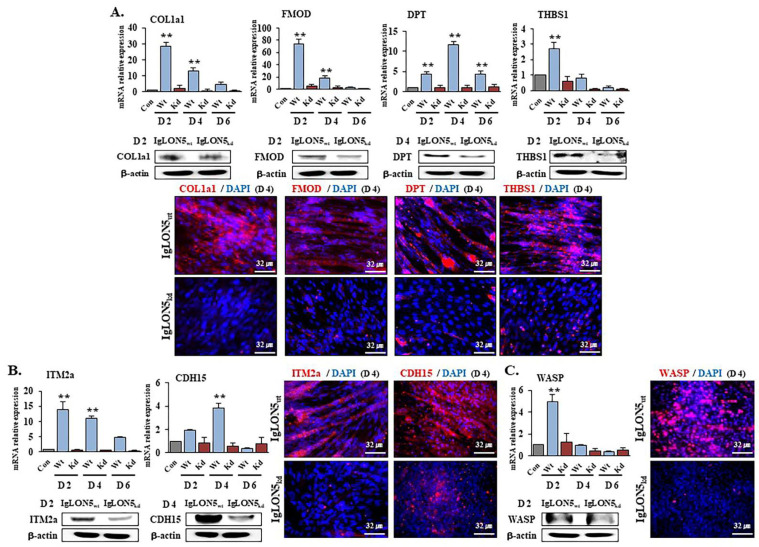
Expressions of myogenic related genes in IgLON5_wt_ and IgLON5_kd_ C2C12 cells. IgLON5_wt_ and IgLON5_kd_ cells were cultured in differentiation medium for 0, 2, 4, and 6 days. mRNA and protein expressions of (**A**) COL1a1, FMOD, DPT, and THBS1, (**B**) ITM2a and CDH15, and (**C**) WASP were determined by real-time RT-PCR, Western blot, and immunocytochemistry. Controls are baseline values. Wt indicates cells transfected with scrambled vector. Means ± SD (n ≥ 3). ** *p* < 0.01. (Scale bar, 32 µm).

To elucidate the mechanism whereby IgLON5 regulates myoblast cell differentiation, we examined gene expressions in IgLON5_wt_ cells and IgLON5_kd_ cells. IgLON5_kd_ cells showed downregulation of ECM components (COL1a1, FMOD, DPT, THBS1) (Figure 4A), membrane proteins (ITM2a, CDH15) (Figure 4B), and a cytoskeletal protein (WASP) (Figure 4C), which confirmed that IgLON5 expression affects the genes expressions of ECM components and the membrane and cytoskeletal proteins required for normal myogenesis.

### 3.5. IgLON5 Expressions in FMOD, DPT, COL1a1, and ITM2a Knockdown Cells

**Figure 5:**
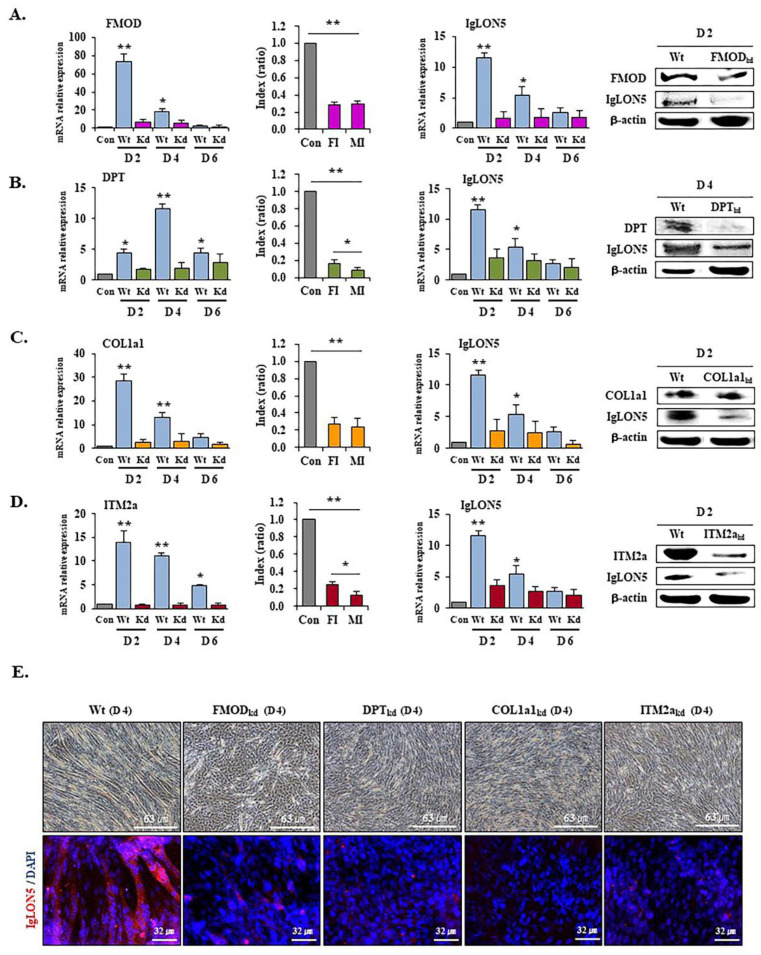
IgLON5 gene expressions in FMOD_kd_, DPT_kd_, COL1a1_kd_, and ITM2a_kd_ C2C12 cells during differentiation. FMOD, DPT, COL1a1, ITM2a mRNA expressions, fusion indices, IgLON5 mRNA and protein expressions in FMOD_kd_ (**A**), DPT_kd_ (**B**), COL1a1_kd_ (**C**), and ITM2a_kd_ (**D**) cells during differentiation as analyzed by real-time RT-PCR, Giemsa staining, and Western blot. Plots on the left in (**A**–**D**) represent relative values of the mRNA expressions in FMOD_kd_, DPT_kd_, COL1a1_kd_, and ITM2a_kd_ cells. (**E**) Localizations of IgLON5 protein in scrambled vector-transfected [31], FMOD_kd_, DPT_kd_, COL1a1_kd_, and ITM2a_kd_ cells on differentiation day 4. Control indicates the day 0 value. FMOD_wt_, DPT_wt_, COL1a1_wt_, and ITM2a_wt_ indicate cells transfected cells with scrambled vector. Means ± SD (n ≥ 3). * *p* < 0.05, ** *p* < 0.01. (Scale bar, 63 or 32 µm).

IgLON5 expressions and fusion indices were significantly less for FMOD_kd_ (FMOD knockdown), DPT_kd_ (DPT knockdown), COL1a1_kd_ (COL1a1 knockdown)_,_ and ITM2a_kd_ (ITM2a knockdown) cells than wild type cells. Interestingly, myotube index reductions were significantly greater than fusion index reductions for DPT_kd_, and ITM2a_kd_ cells, but not for FMOD_kd_ and COL1a1_kd_ cells (Figure 5 and Figure S3). These results support the notion that these four genes are involved in the regulation of IgLON5 expression and myotube formation, and that DPT and ITM2a are more associated with myotube maturation than FMOD or COL1a1.

### 3.6. IgLON5 Expression during Muscle Regeneration

**Figure 6:**
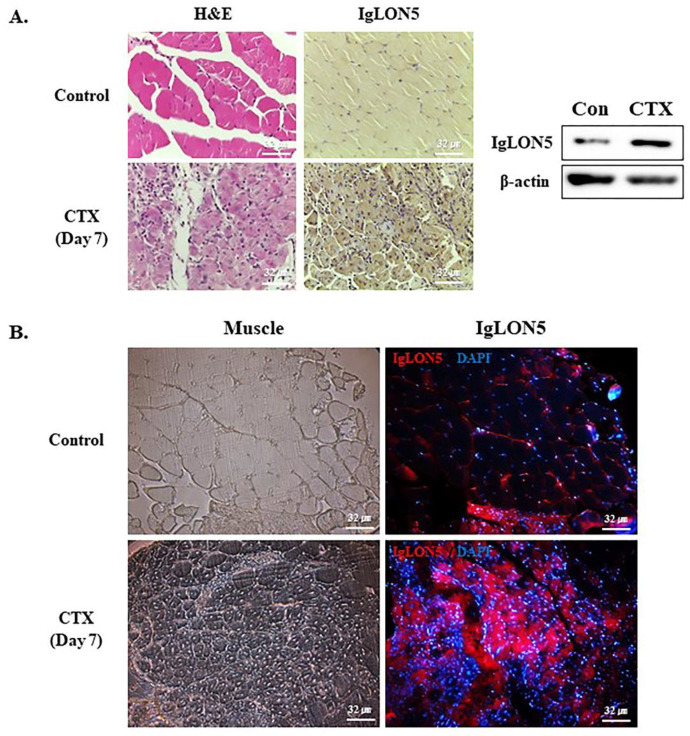
IgLON5 protein expression during muscle regeneration in mice. (**A**) Immunolocalization and protein expression of IgLON5 in the muscles of control and CTX- injected mice 7 days after injection as determined by immunohistochemistry and Western blot (**B**) Immunolocalization expression of IgLON5 in the muscles of control and CTX- injected mice 7 days after injection as determined by Immunofluorescence. (Scale bar, 32 µm).

To investigate the role of IgLON5 in muscle regeneration, IgLON5 protein expression in CTX injected mouse muscles (7 and 14 days) was compared with non-injected muscles (controls). IgLON5 protein levels were greater in CTX injected mice muscles than in controls, (Figure 6A, Figure S4). In addition, IgLON5 expression was detected in control and CTX-injected muscle tissues by immunofluorescence, IgLON5 expression was observed on the membrane of the control muscle tissues, and the CTX-injected muscle undergoing regeneration showed higer IgLON5 expression compared to the control muscles (Figure 6B). Pax7 (MSCs marker) and IgLON5 proteins were simultaneously expressed in 7 days CTX injected muscles (Figure S5). Based on the findings, it was confirmed that IgLON5 was expressed not only in muscle bundles during muscle regeneration, but also in MSCs and in the entire process of muscle regeneration.

## 4. Discussion

Myogenic stem cells (MSCs) proliferate in vitro and fuse to form myotubes, which are the most basic functional units in skeletal muscle tissue and reminiscent of myofibers in vivo [32,33,34]. During myotube formation, MSCs attach to the surfaces of cell culture dishes and communicate with neighboring cells, which is essential for cell survival and myotube formation [2,35]. In our previous study, the IgLON5 gene was found to be markedly downregulated in FMOD_kd_ C2C12 cells during myogenic differentiation [8]. The product of this gene is a cell surface protein and member of the immunoglobulin superfamily and has been shown to be involved in neuronal cell adhesion [23]. However, no study has addressed the involvement of IgLON5 in the proliferation and differentiation of MSCs.

In the present study, we investigated the gene expression patterns of IgLON5 and the mechanisms by which IgLON5 is involved in the proliferation and differentiation of C2C12 cells. We observed that during the transition from cell adhesion to differentiation, IgLON5 gene expression gradually increased and that IgLON5 protein eventually accumulated on the membranes of newly formed myotubes. Knockdown of the IgLON5 gene in C2C12 cells resulted in significant reductions in myotube formation and the expressions of myogenic markers (MYOD, MYOG, and MYL2). Similar results were obtained when wild-type cells were treated with IgLON5-specific antibody, which suggested IgLON5 controlled C2C12 differentiation. Notably, the expressions of the IgLON5 mRNA and protein were increased after this antibody treatment. We speculate that when IgLON5 protein on the surfaces of C2C12 cells was blocked by antibody, a compensatory intracellular signal upregulated IgLON5 gene expression. Interestingly, our observations showed that an increase in IgLON5 protein expression affected differentiation but not proliferation (Figure 1). In fact, percentages of cells in each stage of the cell cycle, glucose uptake, and metabolites (lactate, NH_3_) production were similar in proliferating IgLON5_wt_ and IgLON5_kd_ cells (Figure S2).

Observations of IgLON5 expression increases during differentiation and of much reduced myotube formation by IgLON5_kd_ C2C12 cells, raised the following two questions: 1) What is the molecular mechanism responsible for IgLON5 expression during differentiation? and 2) What is the function of IgLON5 during differentiation? In an experiment where C2C12 cells were monitored by GFP fluorescence, only cells that normally express IgLON5 formed myotubes, affecting the maturation of myotubes (Figure 2). In another experiment, when IgLON5_wt_ or IgLON5_kd_ C2C12 cells cultured in dishes placed inverted and centrifuged with a swinging bucket rotor, IgLON5_wt_ cells adhered better to surfaces of culture dishes and more tightly bound to neighboring cells (Figure 3). This indicated IgLON5 is involved in C2C12 cell adhesion, myotube formation, and maturation during differentiation, which concurs with a previous study, in which it was suggested IgLON5 plays an important role in neuronal cell adhesion [36].

To investigate in more depth the mechanism whereby IgLON5 regulates C2C12 differentiation, we examined the expressions of genes believed to be involved in myogenesis in IgLON5_kd_, FMOD_kd_, DPT_kd_, Cola1_kd_, or ITM2a_kd_ cells and compared these with IgLON5_wt_ cells (Figure 4 and Figure 5). Interestingly, all seven genes examined in IgLON5_kd_ cells were downregulated as compared with IgLON5_wt_ cells, and IgLON5 gene expression was also downregulated in FMOD_kd_, DPT_kd_, Cola1_kd_, and ITM2a_kd_ cells. These results suggest these genes are involved in the regulation of IgLON5 gene expression and myotube formation, and as mentioned above, DPT and ITM2a appear to be more associated with myotube maturation than FMOD or COL1a1. In previous studies [1,8,11], we observed that several genes including DPT and FMOD influence each other’s expressions. We hypothesize that during myogenesis, the abilities of these genes to regulate each other is a considerable advantage as it facilitates complementary activities. in vitro knockdown studies have also shown some genes have profound effects on myogenesis, though in a mouse knockout model, no dramatic effect was observed [37,38]. We believe that this inconsistency between in vitro and in vivo results was caused by microenvironmental differences. In addition, in the case of genes essential for the survival of organisms, another appropriate explanation would be “gene redundancy”, where several genes with similar functions exist.

**Figure 7:**
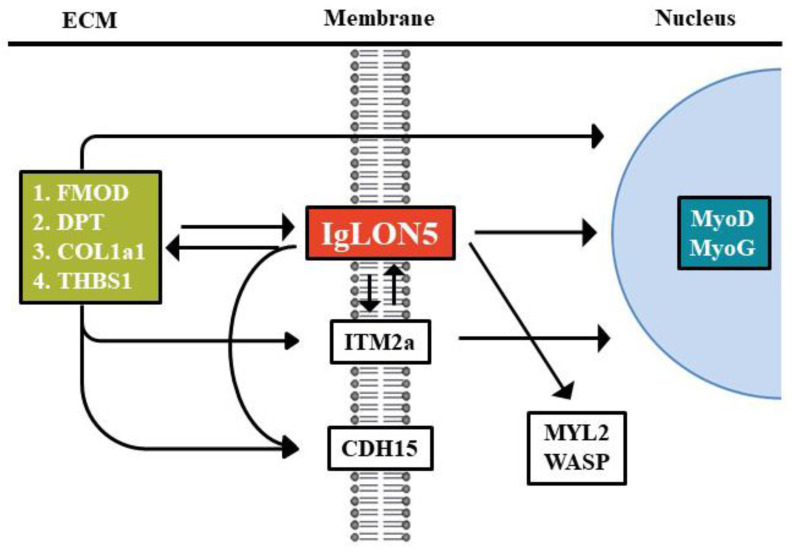
Proposed involvement of IgLON5 in myoblast differentiation.

IgLON5 protein levels were higher in injured muscles than in non-injured muscles (Figure 6A,B and Figures S4 and S5). Our in vitro and in vivo studies provided evidence that IgLON5 is involved in myogenesis, muscle regeneration, and our previous studies and this study show that during myogenesis, myoblasts produce a variety of ECM proteins that presumably create a microenvironment commensurate with proliferation and differentiation and a range of membrane proteins that relay signals of environmental change from cell surfaces to cell interiors. Furthermore, it appears ECM and membrane proteins may control each other’s expressions and compensate for functional losses to create/maintain microenvironments required for myotube formation, maturation, and maintenance (Figure 7).

## Data Availability

The data presented in this study are available in this article and the accompanying Supplementary Material.

## Supplementary Materials

The following are available online at https://www.mdpi.com/2073-4409/10/2/417/s1, Figure S1. Proteins expression by Western blot. IgLON5 knockdown was performed and incubated with differentiation media for 2, 4, and 6 days. Proteins expression was performed by Western blot. Figure S2: Cell cycle and metabolites analysis. Figure S3. Proteins expression by Western blot. Knockdown of FMO, DPT, COL1a1, and ITM2a was carried out and incubated with differentiation media for 2, 4, and 6 days. Proteins expression was analyzed by Western blot. Figure S4. IgLON5 protein expression by immunohistochemistry in CTX injected muscle tissues. IgLON5 Immunolocalization and protein expression was determined by immunohistochemistry in the control muscles tissue and CTX- injected mice muscles tissue at 7 and 14 days. Figure S5. Pax7 and IgLON5 proteins expression by Immunofluorescence at 7 days CTX injecte muscle. Table S1: shRNA sequence information, Table S2: List of PCR primers used.

## Abbreviations

MSCs	myogenic stem (satellite) cells
ECM	Extracellular matrix
IgSF	Immunoglobulin superfamily
OPCML	Opioid-binding cell adhesion molecule
GPI	Glycosylphosphatidylinositol
LSAMP	limbic system-associated membrane protein,
IgLON5	Immunoglobulin-like cell adhesion molecule 5
MYOG	Myogenin
MYOD	Myoblast determination protein
MYL2	Myosin light chain 2
PSP	Progressive supranuclear palsy
FMOD	Fibromodulin
DPT	Dermatopontin
*Itm2A*	*Integral membrane protein 2A*
THBS1	Thrombospondin-1
COL1a1	Collagen type 1 alpha 1
WASP	Wiskott–Aldrich syndrome protein
CDH15	Cadherin 15
CTX	Cardiotoxin
