# Supplementary material for: IgLON5 Regulates the Adhesion and Differentiation of Myoblasts
Source: Cells. 2021 Feb 17;10(2):417. doi: 10.3390/cells10020417 (PMC7922608; doi:10.3390/cells10020417)

Table S1.

Gene	Catalog #	Hairpin sequence (5′– 3′)	Corresponding shRNA (5′– 3′)
**FMOD shRNA Plasmid (m) is a pool of 3 different shRNA plasmids**			
**FMOD**	sc-44823-SHA	GATCCCTACATGGCAACCAGATTATTCAAGAGATAATCTGGTTGCCATGTAGTTTTT	Sense: CUACAUGGCAACCAGAUUAtt<br>Antisense: UAAUCUGGUUGCCAUGUAGtt
	sc-44823-SHB	GATCCCTACTAGACCTGAGTTATATTCAAGAGATATAACTCAGGTCTAGTAGTTTTT	Sense: CUACUAGACCUGAGUUAUAtt<br>Antisense: UAUAACUCAGGUCUAGUAGtt
	sc-44823-SHC	GATCCGCAACAGGATCAATGAGTTTCAAGAGAAACTCATTGATCCTGTTGCTTTTT	Sense: GCAACAGGAUCAAUGAGUUtt<br>Antisense: AACUCAUUGAUCCUGUUGCtt
**DPT shRNA Plasmid (m) is a pool of 3 different shRNA plasmids**			
**DPT**	sc-143012-SHA	GATCCCTGACAGACAATGGAACTATTCAAGAGATAGTTCCATTGTCTGTCAGTTTTT	Sense: CUGACAGACAAUGGAACUAtt<br>Antisense: UAGUUCCAUUGUCUGUCAGtt
	sc-143012-SHB	GATCCCAGTGGAAGTTCATCATGTTTCAAGAGAACATGATGAACTTCCACTGTTTTT	Sense: CAGUGGAAGUUCAUCAUGUtt<br>Antisense: ACAUGAUGAACUUCCACUGtt
	sc-143012-SHC	GATCCCTGTGATTCGCACTATAACTTCAAGAGAGTTATAGTGCGAATCACAGTTTTT	Sense: CUGUGAUUCGCACUAUAACtt<br>Antisense: GUUAUAGUGCGAAUCACAGtt
**Col1a1 shRNA Plasmid (m) is a pool of 3 different shRNA plasmids**			
**Col1a1**	sc-44044-SHA	GATCCCAATGGTGCTCCTGGTATTTTCAAGAGAAATACCAGGAGCACCATTGTTTTT	Sense: CAAUGGUGCUCCUGGUAUUtt<br>Antisense: AAUACCAGGAGCACCAUUGtt
	sc-44044-SHB	GATCCCAAGGTCCTTCTGGATCAATTCAAGAGATTGATCCAGAAGGACCTTGTTTTT	Sense: CAAGGUCCUUCUGGAUCAAtt<br>Antisense: UUGAUCCAGAAGGACCUUGtt
	sc-44044-SHC	GATCCGCAAGACAGTCATCGAATATTCAAGAGATATTCGATGACTGTCTTGCTTTTT	Sense: GCAAGACAGUCAUCGAAUAtt<br>Antisense: UAUUCGAUGACUGUCUUGCtt
**ITM2a shRNA Plasmid (m) is a pool of 3 different shRNA plasmids**			
**ITM2a**	sc-60868-SHA	GATCCCTAGGCCTCTCATTCATCTTTCAAGAGAAGATGAATGAGAGGCCTAGTTTTT	Sense: CUAGGCCUCUCAUUCAUCUtt<br>Antisense: AGAUGAAUGAGAGGCCUAGtt
	sc-60868-SHB	GATCCGGATCCTGTCAATTCCATTTTCAAGAGAAATGGAATTGACAGGATCCTTTTT	Sense: GGAUCCUGUCAAUUCCAUUtt<br>Antisense: AAUGGAAUUGACAGGAUCCtt
	sc-60868-SHC	GATCCCAAGCGTGCCATTGACAAATTCAAGAGATTTGTCAATGGCACGCTTGTTTTT	Sense: CAAGCGUGCCAUUGACAAAtt<br>Antisense: UUUGUCAAUGGCACGCUUGtt
**IgLON5 shRNA Plasmid (m) is a pool of 3 different shRNA plasmids**			
**IgLON5**	sc-140623-SHA	GATCCGGAATACGAATGCGTTACTTTCAAGAGAAGTAACGCATTCGTATTCCTTTTT	Sense: GGAAUACGAAUGCGUUACUtt<br>Antisense: AGUAACGCAUUCGUAUUCCtt

sc-140623-SHB	GATCCCCATCACTGTGAGGGATTATTCAAGAGATAATCCCTCACAGTGATGGTTTTT	Sense: CCAUCACUGUGAGGGAUUAtt<br>Antisense: UAAUCCCUCACAGUGAUGGtt

Table S2.

	Gene	Product size (bp)	Tm (°C)	Forward primer	Reverse primer
**1**	**GAPDH**	155	59	5′- TGC TGG TGC TGA GTA TGT CG - 3′	5′- CAA GCA GTT GGT GGT ACA GG - 3′
**2**	**MYOD**	213	59	5′- AGG AGC ACG CAC ACT TCT CT - 3′	5′- TCT CGA AGG CCT CAT TCA CT - 3′
**3**	**MYOG**	185	59	5′- TCC AGT ACA TTG AGC GCC TA - 3′	5′- CAA ATG ATC TCC TGG GTT GG - 3′
**4**	**MYL2**	177	59	5′- AAA GAG GCT CCA GGT CCA AT - 3′	5′- CCT CTC TGC TTG TGT TGG TCA - 3′
**5**	**IgLON5**	218	59	5′- CTC CGA GAC GGT TTC ACC TC - 3′	5′- ATA GCT TCA CAG CGC AGG AG - 3′
**6**	**FMOD**	155	59	5′- AGG AGC ACG CAC ACT TCT CT - 3′	5′- TCT CGA AGG CCT CAT TCA CT - 3′
**7**	**DPT**	224	59	5′- GGA TCG TGA GTG GCA ATT TT - 3′	5′- CGA ATT CGC AGT CGT AGT CA - 3′
**8**	**COL1a1**	224	59	5′- CTT TGC TTC CCA GAT GTC CT - 3′	5′- CCC CAT CAT CTC CAT TCT TG - 3′
**9**	**ITM2a**	200	59	5′- CAT GCC CAA GAG CAC CAT TT - 3′	5′- AGT CGT GAA TAA TTG CCG CC - 3′
**10**	**THBS1**	162	59	5′- CAT GTG GCAA TGG AAT TCA G - 3′	5′- AAC AGG ACG ACC ATG GAG AC - 3′
**11**	**WASP**	201	59	5′- GCT CCA AAT GGT CCC AAT CT - 3′	5′- CCA TCC AAC ATG CCC AAT GTG - 3′
**12**	**CDH15**	134	59	5′- GGA CTA TGA GAG CCG TGA GC - 3′	5′- GAG CTT CGT TGG TGT CCT GA - 3′

Figure S1.

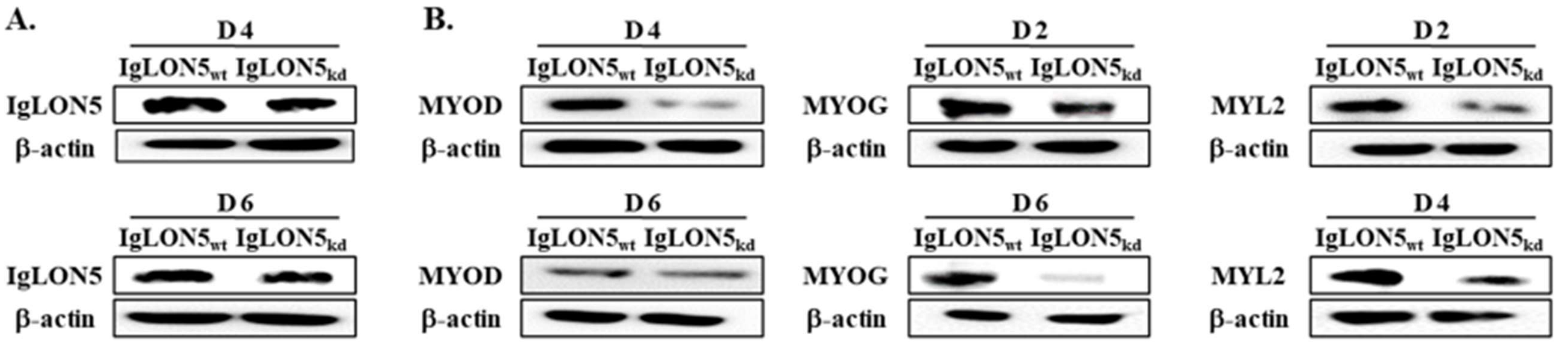

Figure S2.

A.

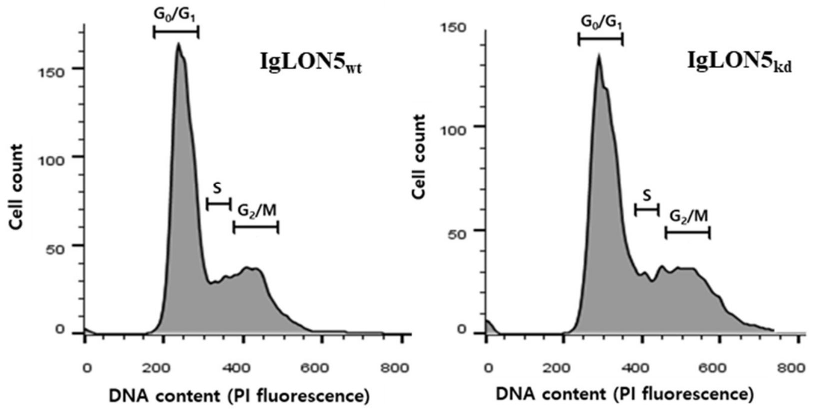

Cell \ Phases	G_0/G_1	S	G_2/M
$IgLON5_{wt}$	59.7% (±3.1%)	21.6% (±2.5%)	16.6% (±2.7%)
$IgLON5_{kd}$	56.8% (±2.5%)	19.8% (±3.4%)	18.4% (±3.7%)

B.

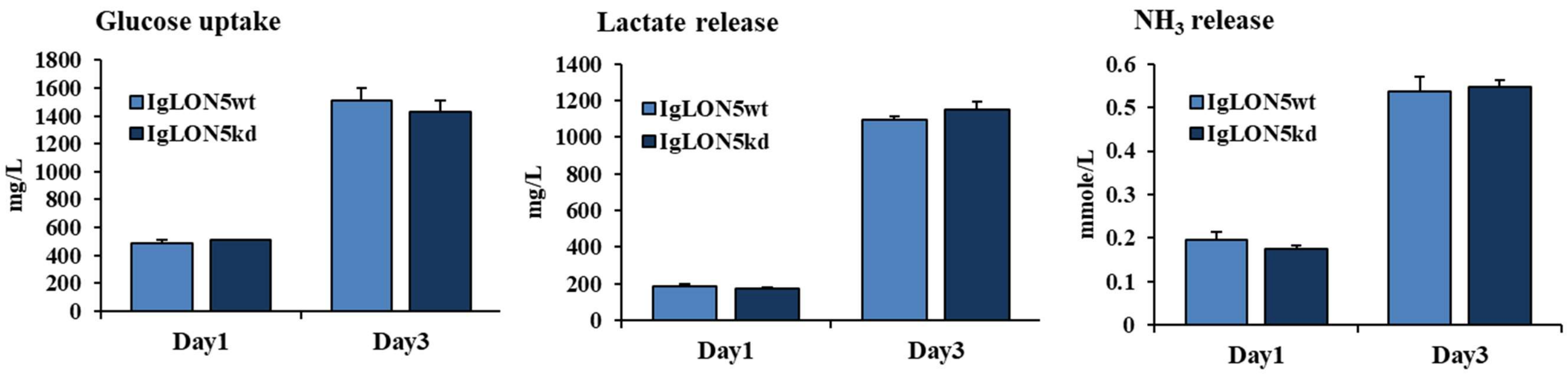

Figure S3.

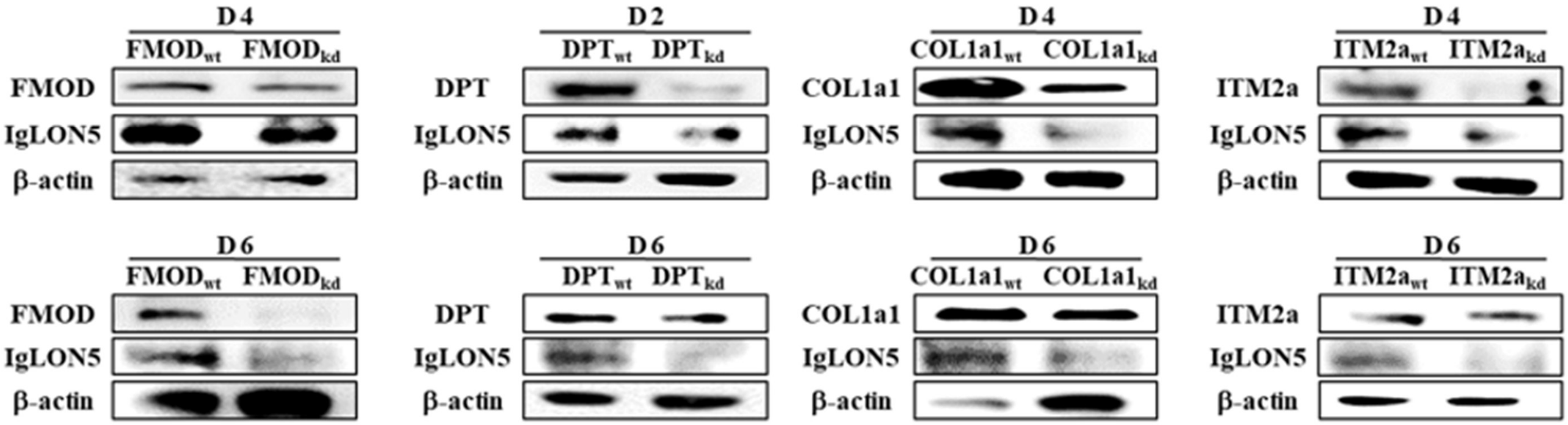
D4
FMODwt FMODkd
FMOD
IgLON5
β-actin
D2
DPTwt DPTkd
DPT
IgLON5
β-actin
D4
COL1a1wt COL1a1kd
COL1a1
IgLON5
β-actin
D4
ITM2awt ITM2akd
ITM2a
IgLON5
β-actin
D6
FMODwt FMODkd
FMOD
IgLON5
β-actin
D6
DPTwt DPTkd
DPT
IgLON5
β-actin
D6
COL1a1wt COL1a1kd
COL1a1
IgLON5
β-actin
D6
ITM2awt ITM2akd
ITM2a
IgLON5
β-actin

Figure S4.

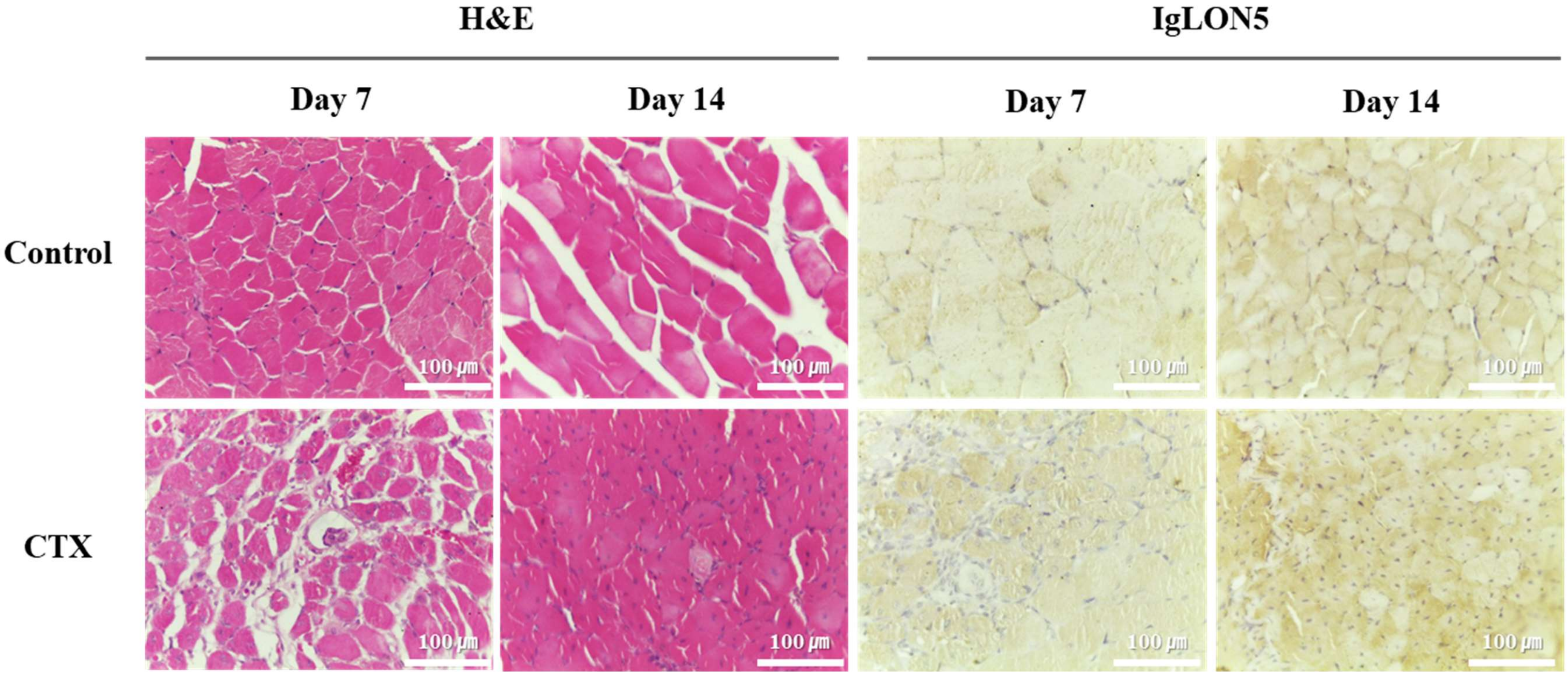
H&E
IgLON5
Day 7
Day 14
Day 7
Day 14
Control
CTX
100 µm
100 µm
100 µm
100 µm
100 µm
100 µm
100 µm
100 µm

Figure S5.

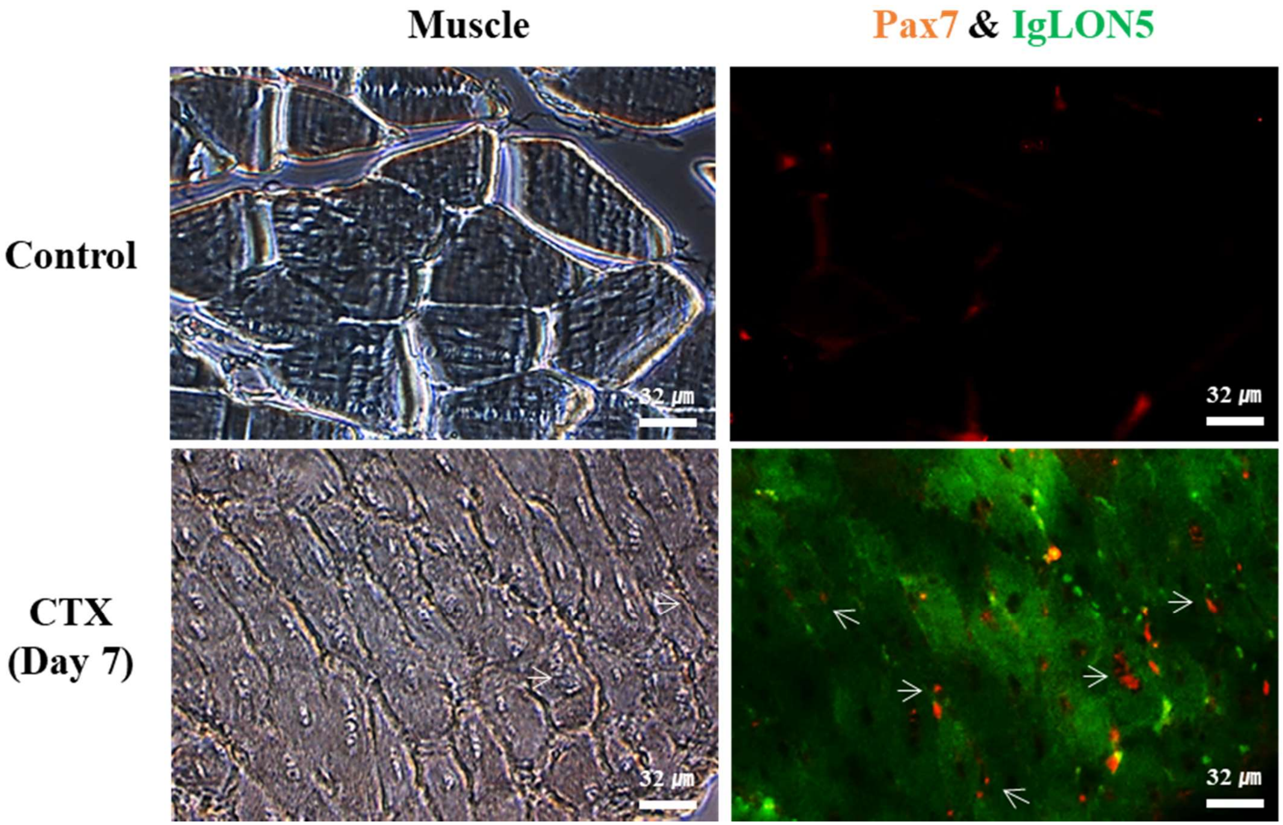

Supplement: Supplementary file 1 [file cells-10-00417-s001.pdf]
